# High prevalence in Malawi of sight-threatening retinopathy and visual impairment caused by diabetes: identification of population-specific targets for intervention

**DOI:** 10.1111/dme.12492

**Published:** 2014-06-07

**Authors:** P I Burgess, T J Allain, M García-Fiñana, N A V Beare, G Msukwa, S P Harding

**Affiliations:** 1 Malawi-Liverpool-Wellcome Trust Clinical Research Programme, Queen Elizabeth Central Hospital, College of MedicineBlantyre, Malawi; 2 Department of Eye and Vision Science, University of LiverpoolLiverpool, UK; 3 Department of Biostatistics, University of LiverpoolLiverpool, UK; 4 Department of St Pauls Eye Unit, Royal Liverpool University HospitalLiverpool, UK; 5 Department of Lions Sight First Eye Hospital, Queen Elizabeth Central HospitalBlantyre, Malawi

## Abstract

**Aims:**

To report the prevalence of all grades of diabetic retinopathy and associations with demographic, clinical and biochemical variables in people with diabetes in Southern Malawi.

**Methods:**

We report baseline data from a 24-month prospective cohort study. Subjects were systematically sampled from two hospital-based, primary care diabetes clinics. Visual acuity, glycaemic control, systolic blood pressure, HIV status, urine albumin–creatinine ratio, and haemoglobin and serum lipid levels were assessed. Retinopathy was graded at an accredited reading centre using modified Wisconsin grading of four-field mydriatic photographs.

**Results:**

A total of 357 subjects were studied. Of these, 13.4% subjects were HIV-positive and 15.1% had anaemia. The overall prevalence rates of any retinopathy, sight-threatening diabetic retinopathy and proliferative retinopathy were 50.1% (95% CI 44.9–55.3), 29.4% (95% CI 24.7–34.1) and 7.3% (95% CI 4.6–10.0), respectively. In multivariate logistic analysis the presence of sight-threatening retinopathy was associated with duration of diabetes (odds ratio 1.11, 95% CI 1.05–1.17), HbA_1c_ (odds ratio 1.31, 95% CI 1.13–1.50), systolic blood pressure (odds ratio 1.03, 95% CI 1.01–1.04), haemoglobin (odds ratio 0.98, 95% CI 0.96–0.99) and LDL cholesterol (odds ratio 1.63, 95% CI 1.18–2.25). No significant association with HIV status was observed. In all, 3.6 and 1.4% of people in our study cohort had visual acuity worse than 6/18 and 6/60 in the better eye, respectively.

**Conclusions:**

The present study found a prevalence of sight-threatening retinopathy in diabetes clinics in one Sub-Saharan African country of approximately four times that reported in recent European studies and a prevalence of proliferative retinopathy approximately 10 times higher. The association of sight-threatening retinopathy with lower haemoglobin level is a new finding. Our results highlight the urgent need for provision of services for retinopathy detection and management to avoid a large burden of vision loss.

**What's new?**

- Global attention is focused on the epidemic of diabetes in Sub-Saharan Africa.
- We provide an estimate of the current prevalence of levels of diabetic retinopathy and visual impairment in people attending diabetes clinics in Malawi.
- The prevalence of sight-threatening diabetic retinopathy and proliferative retinopathy was found to be four and 10 times that reported in recent European studies, respectively.
- We show an association between sight-threatening retinopathy and lower haemoglobin levels: a novel finding and potential therapeutic target.
- Features that differentiate our work from previous cross-sectional studies include the high prevalence of infectious disease (malaria and HIV) and anaemia in our cohort, robust external validation of retinopathy grading at an accredited reading centre, and a comprehensive assessment of systemic variables including HbA_1c_, urine albumin–creatinine ratio and haemoglobin level.

## Introduction

The International Diabetes Federation has estimated that the number of adults with diabetes in Africa will increase from 12.1 million in 2010 to 23.9 million in 2030 1, a presumed consequence of poor diet, sedentary lifestyles, obesity and population growth and aging 2. Diabetes causes visual impairment through early-onset cataracts and diabetic retinopathy, a progressive disease of the retinal microvasculature. The prevalence and incidence of sight-threatening diabetic retinopathy in developed countries have been well documented 3–5. Associations between systemic factors, including glycaemic control 6,7, blood pressure 8 and blood lipid levels 9, and the development and progression of retinopathy in these populations are well known. The epidemiology of diabetic retinopathy in Africa has been systematically reviewed by our group 10. No cohort studies have investigated the determinants of severity and progression of diabetic retinopathy in Sub-Saharan Africa. In this resource-poor setting, population-specific variables, such as a high burden of infectious disease (including HIV and malaria) and anaemia, are likely to affect the spectrum of pathology encountered.

Malawi (population 15.9 million) is one of the poorest countries in Southern Africa, with an annual per capita healthcare expenditure of US$77 11. The recent WHO Malawi national STEPwise survey estimated a prevalence of diabetes of 5.6% in adults 25–64 years, with a similar prevalence in rural and urban areas 12. In 2007, our group performed a pilot, cross-sectional study using clinical ocular examination to assess grades of retinopathy in patients attending the diabetes clinic at Queen Elizabeth Central Hospital, Blantyre 13. That study reported a high prevalence of sight-threatening retinopathy and proliferative retinopathy: 19.6 and 5.7%, respectively.

Because of these important findings, we set out to estimate the prevalence of grades of retinopathy and visual impairment attributable to diabetic retinopathy in a formal observational study using a systematically sampled cohort, standardized clinical photography, independent grading by graders in an accredited reading centre and collection of data on covariates specific to the population. The Malawi Diabetic Retinopathy Study (MDRS) is a prospective, observational, cohort study of patients attending two hospital diabetes clinics over 24 months. The study aims to describe the prevalence, incidence and progression of diabetic retinopathy in Southern Malawi and to investigate the determinants of retinopathy severity and progression in this population. In the present paper, we report baseline data from this cohort.

## Subjects and methods

### Setting

Queen Elizabeth Central Hospital in Blantyre is the main teaching hospital in Malawi. It provides primary and secondary care to the urban and semi-urban population of greater Blantyre (∼1.0 million people, 50% adult), and tertiary care to the southern region (∼ 6.0 million people). Zomba Central Hospital provides primary and secondary care to the urban and rural population of Zomba district. The diabetes clinics at Queen Elizabeth Central Hospital and Zomba Central Hospital provide predominantly primary care level diabetes care and serve ∼2000 and ∼250 patients, respectively.

### Subjects

Systematic random sampling was used to select subjects from the general diabetes clinics at Queen Elizabeth Central Hospital and Zomba Central Hospital (the only public sector diabetes clinics in Blantyre and Zomba) between December 2011 and May 2012. Patients attend these clinics for medical management of diabetes; no eye care is provided. The first subject was selected from the first six people in the diabetes clinic queue using marbles in a bag numbered 1 to 6. Then every sixth individual was approached until 10 subjects were selected (the maximum number of people that could be assessed in a morning). The inclusion criterion was a diagnosis of diabetes according to American Diabetes Association criteria 14. Exclusion criteria were age <18 years, first visit to the diabetes clinic and diagnosis of gestational diabetes according to American Diabetes Association criteria 14. Type 1 diabetes was diagnosed when subjects had commenced insulin therapy within 2 weeks of diagnosis and two of four features were present: age ≤ 19 years at diagnosis; BMI ≤ 25 kg/m^2^; ketones 2 +  on urine analysis; symptoms ≤ 4 weeks duration. Type 2 diabetes was diagnosed in patients stabilized on oral medications or diet from diagnosis (fasting blood sugar ≤7.2 mmol/l on two occasions within 3 months). For subjects not fulfilling the above criteria, a diagnosis of Type 1 or Type 2 diabetes was decided by a clinical panel (P.B. and T.A.). People with Type 2 diabetes were sub-classified based on treatment: insulin with or without oral hypoglycaemic agents, oral hypoglycaemic agents alone or dietary measures alone. The study was approved by the University of Liverpool research ethics committee and the University of Malawi College of Medicine research ethics committee. All participants gave written informed consent.

### Clinical assessment

A standardized pro forma was completed by a nurse by questioning subjects and by reference to the ‘health passport’ carried routinely by patients in Malawi. Physical examination was undertaken by a trained nurse. Blood pressure was measured using the UK Prospective Diabetes Study protocol 8 (HEM-907 XL, OMRON, Lake Forest, IL, USA). Subjects were classified as having hypertension according to the WHO definition 12: subject taking anti-hypertensive medication, or systolic blood pressure ≥140 mmHg, or diastolic blood pressure ≥ 90 mmHg. Weight (Seca 875, Birmingham, UK) and height were recorded.

Visual acuity (uncorrected and using pinhole) was measured as the number of letters read on a standard Early Treatment of Diabetic Retinopathy Study (ETDRS) chart (Sussex Vision, Littlehampton, UK) using a standard protocol (testing at 4 m initially and then at 1 m if <20 letters were read at 4 m). For illiterate subjects, a 4-m log of the minimum angle of resolution ‘Tumbling E’ chart was used (Sussex Vision). For each patient with corrected visual acuity in the better eye of < 80 letters, the primary cause of visual impairment was recorded by the examining clinician (P.B.).

All subjects were offered HIV point-of-care testing according to Malawian national protocol 15 (Determine Rapid Test: Abbott, Hoofddorp, the Netherlands; Uni-Gold: Recombigen, Trinity, Ireland; SD Bioline: Standard Diagnostics, Suwon, Korea). Haemoglobin levels were measured with a point-of-care test (Hb301: HemoCue, Ängelholm, Sweden). Thresholds for anaemia were set according to WHO guidelines: 130 g/l for men; 120 g/l for women 16. Blood samples were assayed for putative biochemical risk factors: fasting glucose, triglycerides, LDL cholesterol, HDL cholesterol, serum creatinine, urine albumin–creatinine ratio (colorimetric assays performed at Malawi Liverpool Wellcome Laboratories, Blantyre, Malawi using the Synchron CX5; Beckman Coulter, Brea, CA, USA) and HbA_1c_ (boronate affinity chromatography performed at Norfolk and Norwich University Hospitals Laboratories, UK).

### Assessment of retinopathy

Retinopathy and maculopathy were classified by feature-specific grading using definitions established in the Liverpool Diabetic Eye Study 17 (Table S1). Macular oedema was assessed according to the ETDRS criteria for clinically significant macular oedema, which is a stage of exudative maculopathy directly threatening or involving the fovea 18. Sight-threatening diabetic retinopathy was defined as any of the following: moderate preproliferative retinopathy or worse (level 40–71 +); macular exudates in a circinate pattern or within one disc diameter of the foveal centre or clinically significant macular oedema (level 3–4: sight-threatening maculopathy); or other diabetes-related retinal vascular disease: central or branch retinal artery occlusion, central or branch retinal vein occlusion.

Digital fundus photography of four 45° standard fields 17 with a stereo macular image was performed through dilated pupils (guttae tropicamide 1% and phenylephrine 2.5%) using CR6 fundus cameras (Canon, Reigate, UK). Dual grading of photographic images was performed by accredited graders at the Liverpool Reading Centre. Additionally, all subjects were examined by one ophthalmologist (P.B.) using slit−lamp biomicroscopy. For the purposes of analysis, if any feature was classified as ‘ungradable’ on photographic grading the biomicroscopy grade for that feature was used.

Biomicroscopy grading was compared with the reference standard of photographic grading. For all grades of retinopathy Cohen's κ was 0.6723 (95% CI 0.606–0.738) and weighted κ 0.820. For grades of maculopathy Cohen's κ was 0.843 (95% CI 0.781–0.905) and weighted κ 0.888 (data not shown). Cataract was graded according to the lens opacities classification system III 19 and considered clinically significant when graded at ≥3 in any category and associated with vision worse than 60 letters (6/18 Snellen).

### Statistical analysis

An *a priori* analysis plan was followed. Grades of retinopathy were calculated by patient according to the worse or only gradable eye. Visual acuity data were investigated by patient according to the better eye and 95% CIs were calculated for proportions. We constructed a logistic regression model (backwards stepwise with probability of removal of 0.2) to determine the odds ratio (OR) and 95% CIs for the presence of sight-threatening retinopathy in association with an initial 11 variables: duration of diabetes, age, sex, systolic blood pressure, HbA_1c_, urine albumin–creatinine ratio, haemoglobin, HIV status, LDL and HDL cholesterol and triglycerides. Adjusted ORs and 95% CIs were calculated for the presence of sight-threatening retinopathy. All tests were two-sided and a *P* value <0.05 was taken to indicate statistical significance. All calculations were performed using stata version 12 (StataCorp, College Station, TX, USA).

## Results

### Participants

A total of 417 people were approached to participate in the study. Of these, 36 declined and 24 were excluded (six did not meet criteria for diagnosis of diabetes; one had gestational diabetes; 17 were either aged < 18 years, visiting the clinic for the first time or resident >60 km from the clinic). A total of 357 people were included (Queen Elizabeth Central Hospital, *n *=* *255; Zomba Central Hospital, *n *=* *102). Participant characteristics are listed in Table1. Of those with Type 2 diabetes, 231 (71.7%) were prescribed oral agents alone, 12 (3.4%) were diet-controlled and 79 (24.5%) were prescribed insulin. A total of 48 (13.4%) subjects were HIV-positive: 34 were taking anti-retroviral therapy; four were HIV-positive but not taking anti-retroviral therapy (all WHO stage 1); and 10 had new diagnoses (WHO stage 1, *n *=* *5; WHO stage 2, *n *=* *2; WHO stage 3, *n *=* *2; and WHO stage 4, *n *=* *1). A total of 17 subjects (4.8%) declined HIV testing. Twenty-four men (17.1%) and 30 women (13.8%) had anaemia. Of the whole cohort 203 subjects were already taking anti-hypertensive medications and an additional 31 were newly diagnosed with hypertension.

**Table 1:** Demographic, clinical and biochemical characteristics of participants in the Malawi Diabetic Retinopathy Study (*N *=* *357)

Characteristic	Entire cohort	Type 1 diabetes	Type 2 diabetes
No. of subjects, *n* (%)	357	35 (9.8)	322 (90.2)
Female gender, *n* (%)	216 (60.5)	8 (22.8)	208 (64.6)
Median (IQR) age, years	54.1 (43.8–61.1)	28.3 (23.1–33.3)	55.2 (47.9–62.2)
Overweight: BMI>25 kg/m^2^, *n* (%)	198 (55.3)	7 (20.0)	191 (59.3)
Median (IQR) time since diagnosis of diabetes, years	4.1 (1.9–8.1)	4.1 (1.4–8.2)	4.1 (2.0–8.1)
Hypertension, *n* (%)	234 (65.5)	3 (8.6)	231 (71.7)
Median (IQR) systolic blood pressure, mmHg	135 (120–156)	116 (109–127)	138 (124–160)
Mean (sd) HbA_1c_			
mmol/l	61.9 (27.5)	81.6 (27.8)	59.8 (26.6)
%	7.8 (2.5)	9.6 (2.5)	7.6 (2.4)
Mean (sd) haemoglobin, g/l	139 (19)	146 (18)	139 (19)
Anaemia, *n* (%)	54 (15.1)	5 (14.3)	49 (15.2)
HIV-positive, *n* (%)	48 (13.4)	4 (11.4)	44 (13.7)
Total cholesterol >5.0 mmol/l, *n* (%)	115 (32.2)	4 (11.4)	111 (34.5)
Mean (sd; range) LDL cholesterol, mmol/l	2.43 (0.95; 0.3–6.0)	1.74 (0.70; 0.6–3.1)	2.51 (0.94; 0.3–6.0)
Raised urine albumin–creatinine ratio: male >2.5 mg/mmol; female >3.5 mg/mmol, *n* (%)	115 (32.2)*	10 (28.6)†	105 (32.6)‡

IQR, interquartile range.

* 51 male; 64 female.

† Seven male; three female).

‡ 44 male; 61 female.

### Prevalence of grades of retinopathy

**Figure 1:**
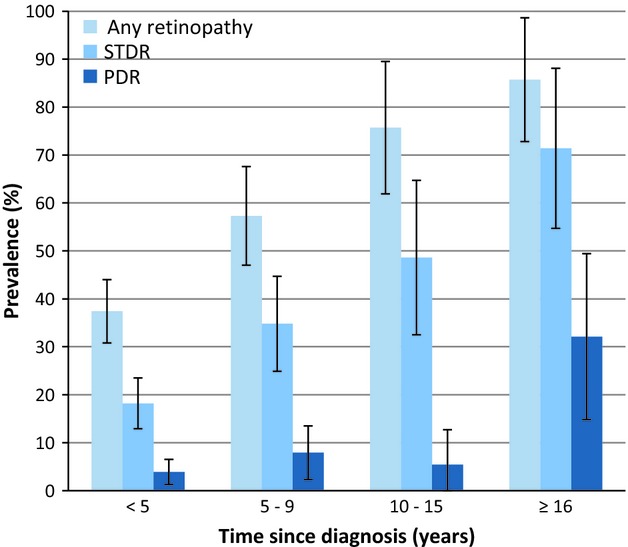
Prevalence of any retinopathy, sight-threatening diabetic retinopathy (STDR) and proliferative diabetic retinopathy (PDR) categorized by time since diagnosis of diabetes in patients in the Malawi Diabetic Retinopathy Study (*N *=* *357).

The prevalence of grades of retinopathy is shown in Table2. The prevalence of retinopathy according to Type 1 or Type 2 diabetes is shown in Table S2. Figure1 shows the prevalence of any retinopathy, sight-threatening retinopathy and proliferative retinopathy categorized by time since diagnosis of diabetes. A total of 25 subjects (7.0%) had cataracts (unilateral, *n *=* *18; bilateral, *n *=* *7) and 16 subjects (4.5%) had pseudophakia (unilateral, *n *=* *5; bilateral, *n *=* *11).

**Table 2:** Prevalence of retinopathy grades according to worse eye among participants in the Malawi Diabetic Retinopathy Study (*N *=* *357)

Grade	Prevalence, *n* (%)	95% CI
No retinopathy (level 10)	177 (49.6)	44.4–54.8
Any retinopathy (level 20–71 + )	179 (50.1)	44.9–55.3
Level 20 retinopathy (haemorrhages or microaneusyms < ETDRS standard photograph 2A)	94 (26.3)	21.8–30.9
Level 30 retinopathy (haemorrhages or microaneusyms ≥ ETDRS standard 2A, and/or 1–6 cotton wool spots)	25 (7.0)	(4.4–9.7)
Level 40 retinopathy (haemorrhages or microaneusyms ≥ ETDRS 2A, and/or ≥6 cotton wool spots, and /or one quadrant venous changes, and/or IRMA < ETDRS standard 8A)	26 (7.3)	4.6–10.0
Level 50 retinopathy (IRMA ≥ ETDRS standard 8A and/or two quadrants venous changes)	8 (2.2)	0.7–3.8
Level 60 or worse (Proliferative retinopathy, fibrovascular proliferation or worse)	26 (7.3)	4.6–10.0
Ungradable	1 (0.3)	0–0.8
Sight-threatening maculopathy (Exudates within one disc diameter of fixation, and/or clinically significant macular oedema, and/or circinate ring of exudates >1 disc area within macula)	93 (26.1)	21.5–30.6
Sight-threatening diabetic retinopathy (Level 40 retinopathy or worse, and/or sight-threatening maculopathy)	105 (29.4)	24.7–34.1

ETDRS, Early Treatment of Diabetic Retinopathy Study; IRMA, intraretinal microvascular abnormalities.

### Associations of retinopathy

Duration of diabetes, HbA_1c_, systolic blood pressure, haemoglobin and LDL cholesterol were risk factors for sight-threatening retinopathy in multivariate analysis (Table3). Descriptive analysis showed that urine albumin–creatinine ratio did not demonstrate a linear association with probability of sight-threatening retinopathy; a natural log transformation was more suitable for the model. An OR of 1.19 for log(albumin–creatinine ratio) corresponds to an OR of 3.29 for urine albumin–creatinine ratio. There was no difference in the prevalence of any retinopathy, sight-threatening retinopathy and proliferative retinopathy between subjects from Blantyre and Zomba (Table S3).

**Table 3:** Risk factors for association of sight-threatening diabetic retinopathy in participants in the Malawi Diabetic Retinopathy Study (*N *=* *357): univariate and multivariate logistic regression

	OR	95% CI	*P*
**Univariate logistic regression**			
Duration of diabetes (years)	1.13	1.08–1.18	0.001*
HbA_1c_ (mmol/mol)	1.01	1.00–1.02	0.004
Systolic blood pressure, mmHg	1.02	1.01–1.03	0.001*
log[urine albumin–creatinine ratio] (mg/mmol)	1.42	1.22–1.65	0.001*
Haemoglobin (g/l)	0.98	0.97-0.99	0.003*
HIV-positive	0.43	0.19–0.95	0.037*
LDL cholesterol (mmol/l)	1.41	1.10–1.80	0.006*
HDL cholesterol (mmol/l)	1.90	0.97–3.73	0.060
Triglycerides (mmol/l)	0.99	0.82–1.21	0.933
Sex (male)	0.61	0.38–0.99	0.045*
Age (years)	1.01	0.99 - 1.03	0.142
**Multivariate logistic regression**			
Duration of diabetes (years)	1.11	1.05–1.17	0.001*
systolic blood pressure (mmHg)	1.03	1.01–1.04	0.001*
HbA_1c_ (mmol/mol)	1.02	1.01–1.04	0.001*
Haemoglobin (g/l)	0.98	0.96–0.99	0.011*
LDL cholesterol (mmol/l)	1.63	1.18–2.25	0.003*
log[urine albumin–creatinine ratio] (mg/mmol)	1.19	0.98–1.44	0.073
Age (years)	0.97	0.95–1.00	0.053

OR, odds ratio.

* *P* < 0.05.

### Treatment

One subject had undergone a course of laser photocoagulation before enrolment in the study and 63 subjects were listed for a course of laser treatment at their first study visit. The threshold for scatter laser treatment was the ‘4-2-1 rule’ (four quadrants of haemorrhages and microaneurysms ≥ ETDRS standard photograph 2A, or two quadrants of venous beading ≥ 6A, or one quadrant of intraretinal microvascular abnormalities ≥8A). The threshold for macular laser treatment was clinically significant macular oedema or exudates tracking towards the foveal centre. A total of 39 subjects were listed for both scatter and macular laser in one or both eyes; 11 subjects were listed for scatter treatment alone; 13 subjects were listed for macular laser alone. Anti-vascular endothelial growth factor agents were not available to study subjects. In all, 25 subjects (7%) had sight-threatening maculopathy (bilateral, *n *=* *9; unilateral, *n *=* *16) and vision < 70 letters (6/12 Snellen).

### Vision

Visual acuity measurements for study subjects are shown in Table4. According to WHO definitions 20, 343 subjects (96.1%; 95% CI 94.1–98.1) had ‘normal vision’ (≥60 letters), eight subjects (2.2%; 95% CI 0.7–3.8) had ‘moderate visual impairment’ (50–59 letters), and five subjects (1.4%; 95% CI 0.2–2.6) were ‘severely visually impaired or blind’ (<50 letters). The most common primary causes of visual impairment for subjects with corrected visual acuity worse than 60 letters (equivalent to 6/18 Snellen or worse) were diabetic retinopathy (46.2%), cataracts (15.4%) and both diabetic retinopathy and cataracts (15.4%) (Table S4). In 61.6% of cases, therefore, diabetic retinopathy was the sole or equal contributing cause of visual loss. In univariate analysis vision < 70 letters was significantly associated with increasing age (OR 1.04; 95%CI 1.01–1.08; *P *=* *0.01), duration of diabetes (OR 1.06; 95% CI 1.01–1.12; *P *=* *0.03) and sight-threatening retinopathy (OR 2.59; 95%CI 1.16–5.79; *P *=* *0.02).

**Table 4:** Prevalence of corrected visual acuities according to better eye in patients in the Malawi Diabetic Retinopathy Study (*N *=* *357)

ETDRS visual acuity*	*n*	95% CI
≥ 90 (6/5)	88 (24.6)	20.1–29.1
80–89 (6/7.5)	171 (47.9)	42.7–53.1
70–79 (6/12)	71 (19.9)	15.8–24.0
60–69 (6/18)	13 (3.6)	1.7–5.6
50–59 (6/30)	8 (2.2)	0.7–3.7
40–49 (6/75)	3 (0.8)	0–1.7
Hand movements	1 (0.3)	0–0.8
Light perception	1 (0.3)	0–0.8
No light perception	0 (0)	
No data	1 (0.3)	0–0.8

* Approximate Snellen acuities are provided in parentheses.

ETDRS, Early Treatment of Diabetic Retinopathy Study.

## Discussion

We report the baseline prevalence of diabetic retinopathy and visual impairment as well as associations of sight-threatening retinopathy in our MDRS cohort. Subjects were sampled from a mixed urban and rural population attending clinics for routine primary and secondary diabetes care. We found retinopathy in 50% of our cohort. This was sight-threatening in 30% of people, with immediately sight-threatening proliferative disease in 7.3%. In multivariate analysis, duration of diabetes, worse glycaemic control, higher systolic blood pressure, lower haemoglobin and elevated LDL cholesterol were significantly associated with presence of sight-threatening retinopathy. In this selected population, the prevalence of vision in the better eye of <60 letters (6/18) was 3.6%. In 61.6% of subjects with visual loss, diabetic retinopathy was the sole or equal contributing cause.

The present study found a higher prevalence of any retinopathy, sight-threatening retinopathy and prolifera-tive retinopathy than was reported in our 2007 pilot study (any retinopathy 32.0%; sight-threatening retinopathy 19.6%; proliferative retinopathy 5.7%) 13, which formed part of a larger cross-sectional survey of diabetes complications 21. Higher estimates in the present study may reflect differences in subject sampling (systematic vs *ad hoc*), grading of retinopathy (accredited grading of standard photographs vs clinical grading), differences between centres (this study also included subjects from Zomba, which is a more rural setting), and changes in disease prevalence over time. Two population-based studies from Africa have reported prevalence of retinopathy in people with diabetes, neither of these was from Sub-Saharan Africa. In these two studies from Egypt 22 and Mauritius 23 the prevalence range for any retinopathy was 30.2–31.6%, proliferative retinopathy 0.9–1.3%, and any maculopathy 1.2–4.5%. Clinic-based studies from Sub-Saharan Africa report a wide range of prevalence but vary widely in quality and methods. Very high prevalence rates of diabetic retinopathy, proliferative retinopathy and maculopathy have been reported in clinic-based surveys from South Africa, for example, by Rotchford *et al*. 24 (40.3% any retinopathy, 5.6% proliferative retinopathy, 10.3% clinically significant macular oedema). These estimates are similar to those in the present study, reflecting similarities between these populations in socio-economic status, access to healthcare, diet and levels of infectious and non-communicable comorbidity.

Population-based studies from low- and middle-income countries have reported lower rates. The Chenai Urban Rural Epidemiology Study reported a prevalence of retinopathy of 17.6% in 1736 subjects with Type 2 diabetes 25. In the Snakara Nethralaya Diabetic Retinopathy Epidemiology and Molecular Genetics Study in urban Indian subjects aged > 40 years with diabetes, the prevalence rates of any retinopathy, proliferative retinopathy and clinically significant macular oedema were 18, 1.6 and 1.4%, respectively 26. In Europe the population-based Liverpool Diabetic Eye Study 3 reported that prevalence rates of any retinopathy, sight-threatening retinopathy and proliferative retinopathy were 27.4, 7.0 and 0.8%, respectively, in 8062 subjects with diabetes entering a primary care-based screening programme. The number of subjects with diet-controlled diabetes in the present study was low; however, even after removing diet-controlled subjects from both cohorts, the prevalence rates of sight-threatening retinopathy and proliferative retinopathy in the present study were approximately three times and 10 times higher, respectively, compared with those of the Liverpool Diabetic Eye Study (data not shown). The high prevalence of retinopathy in the present study compared with recent Asian and European studies is probably attributable to late diagnosis of diabetes, poor access to health services and inadequate drug supply, as well as comorbidity.

In common with the present study, the risk of development and progression of retinopathy in European and North American populations has been shown to be related to duration of diabetes 4,5, high HbA_1c_ levels 6,7, high blood pressure 8, serum lipid levels 9 and microalbuminuria 27. We have demonstrated a novel association between lower haemoglobin and the presence of sight-threatening retinopathy. We hypothesize that the mechanism underlying this relationship is impaired oxygen delivery and therefore increased oxygen stress at a microvascular level.

The aetiology of anaemia in Sub-Saharan Africa is multifactorial and includes deficiencies of micronutrients (e.g. iron, B12, folate), haemoglobinopathies, infections and chronic diseases (e.g. malaria, HIV, tuberculosis) 28. Micronutrient deficiencies are potential therapeutic targets. Whether treatment of anaemia reduces diabetic microvascular complications is not known.

A potential confounder of the association between haemoglobin and retinopathy is socio-economic status. Socio-economic data were not collected in the present study. Both HIV infection and anti-retroviral therapies are associated with a vasculopathy which manifests as increased cardiovascular and cerebrovascular risk 29. There is evidence of higher prevalence of diabetic complications in people with HIV 21. This study showed no significant relationship between presence of sight-threatening retinopathy and HIV status. The effect of HIV on diabetic retinopathy progression will be shown by our cohort study.

Few studies have investigated visual acuity in people with diabetes in Sub-Saharan Africa. The prevalence of visual impairment in this study (1.4% of subjects with visual acuity 6/60 or worse in the better eye) is comparable with published European and American data. In the Wisconsin Epidemiological Study of Diabetic Retinopathy, a visual acuity of 6/60 or worse in the better eye occurred in 3.6% of people with Type 1 and 1.6% of people with Type 2 diabetes 30. In Iceland, Kristinsson *et al*. 25 reported visual acuity of 6/60 or worse in 1.6% of people with Type 2 diabetes. The similar levels of visual impairment are surprising, given the higher prevalence of sight-threatening retinopathy in the present cohort. A potential bias could be that subjects who become visually impaired may cease to attend clinics or die prematurely. If this is the case, our results show that a high proportion of patients attending clinics have (potentially treatable) sight-threatening retinopathy which is not yet symptomatic. In the present study, 63 people were listed for a course of laser treatment while only one person had received laser treatment before the study. This equates to a laser coverage of 1.6% at the time of the study.

Our findings are likely to be representative of small cities/large towns in Sub-Saharan Africa but should be generalized to other settings with caution. While some patients travel long distances to attend clinics, rural subjects are likely to be under-represented. It is possible that our data underestimate retinopathy. Patients who do not attend clinics may be less likely to be diagnosed with diabetes or to comply with therapy. Conversely, those with established complications may be more likely to attend clinics and participate in research studies.

In the present study we have provided an estimate of the current prevalence of diabetic retinopathy and visual impairment in a mixed urban and rural population attending primary care diabetes clinics in Sub-Saharan Africa. We have shown a novel association of sight-threatening retinopathy, haemoglobin level, and have reported the number of people requiring laser treatment. The prevalence of diabetes in Africa is increasing rapidly and there is an urgent need for service provision. This study provides data which are vital for the design of prevention and early detection programmes in the region. Our findings represent a baseline against which the efficacy and cost-effectiveness of such interventions can be judged.

## Funding sources

This work was funded by the Wellcome Trust via a Clinical PhD Fellowship (P.B. Grant number 094015/Z/10/A). The funding body had no role in study design, data collection and analysis, decision to publish, or preparation of the manuscript.

## Competing interests

None declared.

## Supporting Information

Table S1 Levels of retinopathy and maculopathy in the Liverpool Diabetic Eye Study **Table S2** Prevalence with 95% CI of retinopathy grades according to worse eye in patients with Type 1 (*n *=* *35) and Type 2 diabetes (*n *=* *322) **Table S3** Prevalence of retinopathy grades according to worse eye in 357 subjects in the Malawi Diabetic Retinopathy Study, categorized by location **Table S4** Primary causes of visual impairment in the opinion of the examining clinician for subjects with corrected visual acuity equivalent to 6/12 Snellen or worse. Subjects classified according to level of visual impairment (*n *=* *97)
