# Supplementary material for: High prevalence in Malawi of sight-threatening retinopathy and visual impairment caused by diabetes: identification of population-specific targets for intervention
Source: Diabet Med. 2014 Jun 7;31(12):1643–50. doi: 10.1111/dme.12492 (PMC4257078; doi:10.1111/dme.12492)
Supplement: Table S1 — Levels of retinopathy and maculopathy in the Liverpool Diabetic Eye Study Table S2 Prevalence with 95% CI of retinopathy grades according to worse eye in patients with Type 1 (n = 35) and Type 2 diabetes (n = 322) Table S3 Prevalence of retinopathy grades according to worse eye in 357 subjects in the Malawi Diabetic Retinopathy Study, categorized by location Table S4 Primary causes of visual impairment in the opinion of the examining clinician for subjects with corrected visual acuity equivalent to 6/12 Snellen or worse. Subjects classified according to level of visual impairment (n = 97) [file dme0031-1643-SD1.docx]

**ELECTRONIC SUPPLEMENTARY MATERIAL**

**ESM Table 1**. Levels of retinopathy and maculopathy in the Liverpool Diabetic Eye Study

**Level Definition**

**Retinopathy**

1. No retinopathy

20 Haemorrhages or microaneurysms < ETDRS standard photograph 2A †

30 Haemorrhages or microaneurysms ≥ ETDRS standard photograph 2A, and/or 1-6 cotton wool spots

40 Haemorrhages/ microaneurysms ≥ ETDRS STD 2A

and/or ≥ 6 CWS

and/or 1 quadrant venous changes

and/or IRMA < ETDRS STD 8A

50 IRMA ≥ ETDRS STD 8A

and/or 2 or more quadrants venous changes

and/or pre-retinal haemorrhage in absence of proliferation

60 Fibrovascular proliferation

and/or proliferative retinopathy

70 Diabetic Retinopathy Study high risk characteristics

71 Tractional retinal detachment

72 No fundal view due to vitreous blood

90 Ungradeable due to any other reason e.g. media opacity

**Maculopathy**

0 No maculopathy

1 Questionable: < 50% certainty of presence of exudate

2 Exudate >1 disc diameter from fixation

3 Circinate ring of exudate within macula >1 disc area in size but not within 1 disc diameter of fixation

4 Exudates within 1 disc diameter of fixation

and/or presence of clinically significant macular oedema

8 Exudates due to other diseases e.g. vein occlusion, CNV

90 Ungradeable

† Definition of any diabetic retinopathy: ≥ 1 HMa in either eye. Flame shaped hemorrhages associated with hypertension are discounted. IRMA = intraretinal microvascular abnormalities. ETDRS = Early Treatment Diabetic Retinopathy Study

**ESM Table 2.** Prevalence with 95% CI of retinopathy grades according to worse eye in patients with type 1 (n=35) and type 2 diabetes (n=322).

|  | **Type 1 diabetes** | | **Type 2 diabetes** | |
| --- | --- | --- | --- | --- |
| **Grade** | **n** | **% (95% CI)** | **n** | **% (95% CI)** |
| **No retinopathy (level 10)** | 20 | 57.1 (40.8 – 73.5) | 157 | 48.8 (43.3 – 54.2) |
| **Any retinopathy (level 20-71+)** | 15 | 42.9 (26.5 – 59.3) | 164 | 50.9 (45.5 – 56.4) |
| **Level 20 retinopathy** | 10 | 28.6 (13.6 - 43.5) | 84 | 26.1 (21.3 – 30.9) |
| **Level 30 retinopathy** | 1 | 2.9 (0 – 8.4) | 24 | 7.5 (4.6 – 10.3) |
| **Level 40 retinopathy** | 1 | 2.9 (0 – 8.4) | 25 | 7.8 (4.8 – 10.7) |
| **Level 50 retinopathy** | 0 | 0 | 8 | 2.5 (0.8 – 4.2) |
| **Proliferative or worse**  **(≥ level 60 retinopathy)** | 3 | 8.6 (0 – 17.9) | 23 | 7.1 (4.3 – 10.0) |
| **Ungradeable** | 0 | 0 | 1 | 0.3 (0 – 0.9) |
| **Sight threatening maculopathy** | 6 | 17.1 (4.7 – 29.6) | 87 | 27.0 (22.2 – 31.9) |
| **STDR** | 9 | 25.7 (11.2 – 40.2) | 96 | 29.8 (24.8 – 34.8) |

**ESM Table 3.** Prevalence of retinopathy grades according to worse eye in 357 subjects in the MDRS categorised by location.

| **Grade**  (**n; %; 95% CI)** | **Queen Elizabeth Central Hospital, Blantyre** | **Zomba Central Hospital** | p value |
| --- | --- | --- | --- |
| **n** | 255 | 102 |  |
| **No retinopathy**  **(level 10)** | 126 (49.4)  (43.3-55.5) | 51 (50.0)  (40.3-59.7) | p = 0.99  Fisher’s exact |
| **Any retinopathy**  **(level 20-71+)** | 128 (50.2)  (44.1-56.3) | 51 (50.0)  (40.3-59.7) | p = 0.99  Fisher’s exact |
| **Level 20 retinopathy** | 66 (25.9)  (20.5-31.3) | 28 (27.5)  (18.8-36.2) |  |
| **Level 30 retinopathy** | 21 (8.2)  (4.8-11.6) | 4 (3.9)  (0.1-7.7) |  |
| **Level 40 retinopathy** | 17 (6.7)  (3.6-9.8) | 9 (8.8)  (3.3-14.3) |  |
| **Level 50 retinopathy** | 3 (1.2)  (0-2.5) | 5 (4.9)  (0.7-9.1) |  |
| **Proliferative or worse**  **(≥ level 60 retinopathy)** | 21 (8.2)  (4.8-11.6) | 5 (4.9)  (0.7-9.1) | p = 0.37  Fisher’s exact |
| **Ungradable** | 1 (0.4)  (0-1.2) | 0 |  |
| **Sight threatening maculopathy** | 66 (25.9)  (20.5-31.3) | 27 (26.5)  (17.9-35.1) |  |
| **Sight threatening diabetic retinopathy** | 73 (28.6)  (23.1-34.2) | 32 (31.4)  (22.4-40.4) | p = 0.61  Fisher’s exact |

**ESM Table 4.** Primary causes of visual impairment in the opinion of the examining clinician for subjects with corrected visual acuity equivalent to 6/12 Snellen or worse. Subjects classified according to level of visual impairment (n=97).

|  | **Level of visual impairment** | | | |
| --- | --- | --- | --- | --- |
|  | **6/12** | **6/18** | **Moderate visual impairment** | **Severely visually impaired or blind** |
| **n** | 71 | 13 | 8 | 5 |
| **DR** | 24 (34%) | 2 (15%) | 5 | 1 |
| **DR and cataract** | 9 (13%) | 4 (31%) | 1 | 1 |
| **Cataract** | 20 (28%) | 6 (46%) | 1 | 1 |
| **AMD** | 3 (4%) |  |  |  |
| **Glaucoma** | 2 (3%) |  |  |  |
| **Other** | 13* (18%) | 1§ (8%) | 1≠ | 2ƚ |

Details of other causes of visual impairment:

***** 1 Posterior capsule opacification (PCO); 2 Epiretinal membrane; 1 optic atrophy; 1 complicated cataract surgery; 1 dry eye; 1 macular hole; 1 unidentified maculopathy; 5 no cause identified.

§ PCO

≠ Optic atrophy secondary to sphenoid meningioma

Ƚ 1 inherited retinopathy; 1 posterior uveitis (likely syphilitic)
